# Malnutrition management of hospitalized patients with diabetes/hyperglycemia and COVID-19 infection

**DOI:** 10.1007/s11154-022-09714-z

**Published:** 2022-03-04

**Authors:** Rosa Burgos, José Manuel García-Almeida, Pilar Matía-Martín, Samara Palma, Alejandro Sanz-Paris, Ana Zugasti, José Joaquín Alfaro, Ana Artero Fullana, Alfonso Calañas Continente, María Jesús Chicetru, Katherine García Malpartida, Ángela González Faes, Víctor González Sánchez, María Lainez López, Antonio Jesús Martínez Ortega, Juana Oliva Roldán, Clara Serrano Moreno, Pablo Suárez Llanos

**Affiliations:** 1 Unidad de Soporte Nutricional, Hospital Universitari Vall d’Hebron, Barcelona, Spain; 2 Unidad de Gestión Clínica de Endocrinología Y Nutrición, Hospital Virgen de La Victoria, Málaga, Spain; 3 Departamento de Endocrinología Y Nutrición, Hospital Clínico San Carlos, Madrid, Spain; 4 Unidad de Nutrición Clínica Y Dietética, Hospital Universitario de La Paz, Madrid, Spain; 5 Nutrition Department, Miguel Servet University Hospital, 50009 Zaragoza, Spain; 6 Instituto de Investigación Sanitaria (IIS) Aragón, 50009 Zaragoza, Spain; 7 Unidad de Nutrición Clínica, Hospital Universitario de Navarra, 31008 Pamplona, Spain; 8 Complejo Hospitalario Universitario de Albacete, Albacete, Spain; 9 Hospital Universitario General, Valencia, Spain; 10 Hospital Universitario Reina Sofía, Córdoba, Spain; 11 Hospital San Pedro, La Rioja, Spain; 12 Hospital Universitario Politécnico La Fé, Valencia, Spain; 13 Hospital de Marqués de Valdecilla, Santander, Spain; 14 Hospital de Alcorcón, Madrid, Spain; 15 Hospital Juan Ramon Jimenez, Huelva, Spain; 16 Hospital Universitario Torrecardenas, Huelva, Spain; 17 Hospital Universitario Infanta Sofía, Madrid, Spain; 18 Hospital Universitario Gregorio Marañón, Madrid, Spain; 19 Hospital Nuestra Señora de La Candelaria, Tenerife, Spain

**Keywords:** COVID-19, Diabetes, Hyperglycemia, Malnutrition, Medical nutrition

## Abstract

Diabetes mellitus and/or hyperglycemia are highly prevalent medical conditions in patients hospitalized for coronavirus disease 2019 (COVID-19) and are associated with adverse outcomes. In addition, COVID-19 itself can provoke fluctuating and high glucose levels that can be difficult to manage upon hospitalization**.** Hospitalized patients with COVID-19 are at high risk of malnutrition due to an increase in nutritional requirements and a severe acute inflammatory response. The management of patients with diabetes/hyperglycemia and COVID-19 is challenging and requires a specific nutritional approach, the purpose of which is to fulfill the nutritional requirements while maintaining an optimal glycemic control. In this study, an expert group of nutritional endocrinologists carried out a qualitative literature review and provided recommendations based on evidence and guidelines, when available, or on their own experience. The optimal care based on these recommendations was compared with the routine bedside care as reported by a panel of physicians (mainly, endocrinologists, geriatricians, and internists) treating patients with diabetes/hyperglycemia and COVID-19 in their daily practice. Early screening and diagnosis, a diabetes-specific therapeutic approach, and a close malnutrition monitoring are essential to improve the clinical outcomes of these patients. In conclusion, the proposed recommendations are intended to provide a useful guide on the clinical management of malnutrition in patients with COVID-19 and diabetes/hyperglycemia, in order to improve their outcomes and accelerate their recovery. The comparison of the recommended optimal care with routine clinical practice could aid to identify gaps in knowledge, implementation difficulties, and areas for improvement in the management of malnutrition in this population.

## Introduction

The coronavirus disease 2019 (COVID-19), declared a global pandemic by the World Health Organization in March 2020, is caused by the severe acute respiratory syndrome coronavirus 2 (SARS-CoV-2), and has provoked an excess morbidity and mortality and a great public health burden worldwide [1, 2]. The clinical manifestations of COVID-19 are unspecific and similar to many other diseases of viral etiology. The most frequent symptoms are fever, cough, headache, fatigue, anorexia, and myalgias [3, 4], while anosmia and dysgeusia are somewhat less frequent but more specific, although not exclusive, of COVID-19 [5]. In patients requiring hospitalization, COVID-19 is characterized by dyspnea, hypoxemia, and bilateral pneumonia, which can lead to severe respiratory failure requiring admission to intensive care units (ICU) [6, 7]. In these cases, organ dysfunctions are common, mainly due to lung and cardiovascular injury. Recently, a direct injury on insulin-secreting β cells in the pancreas has been described [8].

Together with hypertension, diabetes mellitus and/or hyperglycemia are highly prevalent medical conditions in patients hospitalized for COVID-19 [6, 9] and have demonstrated to be poor prognostic factors, increasing the probabilities of ICU admission and mortality [10, 11]. People with diabetes experiment several pathophysiological changes that could make them prone to a more severe COVID-19, including chronic inflammation, increased coagulation activity, compromised immune response, endothelial dysfunction, and potential pancreatic damage by SARS-CoV-2 [12, 13], whereas sustained hyperglycemia has been associated with persistently elevated interleucin-6 and D-dimer levels during hospitalization [14]. Furthermore, the COVID-19 itself can provoke fluctuating and high glucose levels that can be difficult to manage upon hospitalization and are associated with worse outcomes [15].

Chronic and acute infections negatively impact on nutritional state. Disease-related malnutrition (DRM) is a condition to fight in any disease that requires hospital admission, since it is a poor prognostic factor [16, 17]. Hospitalized patients with COVID-19 are at high risk of DRM due to an increase in nutritional requirements and the severe acute inflammatory response that they usually develop [18, 19]. In addition, various COVID-19 symptoms can compromise a sufficient intake, such as anorexia, dyspnea, asthenia, ageusia/anosmia, cough and dysphagia, whereas digestive symptoms (bloating or abdominal pain, diarrhea) entail abnormal absorption of nutrients [20]. Thus, the management of these patients is challenging and requires a specific nutritional approach, the purpose of which is to fulfill the nutritional requirements while maintaining an optimal glycemic control [21]. The objectives of this study were to generate, based on the available data and clinical experience, consensus recommendations by a group of Spanish experts for the clinical management of malnutrition in hospitalized patients with COVID-19 and diabetes/hyperglycemia, and to compare the optimal care based on these recommendations with the routine bedside care as reported by a panel of Spanish physicians in their daily practice.

## Methods

### Expert group-based consensus recommendations

The scientific committee consisted of 6 coordinators and 12 clinical experts, all selected according to the following criteria: members of the Spanish Society of Endocrinology and Nutrition and/or Chief of their hospitals’ Endocrinology and Nutrition Departments, with more than 10 years of experience in ground and treating hospitalized patients with COVID-19. The 6 coordinators conducted a comprehensive literature search on Pubmed/Medline to identify relevant articles and clinical guidelines. The keywords that were used included malnutrition, diabetes, hyperglycemia, SARS-CoV-2, and COVID-19 (bibliographic search performed in October 2020, updated in October 2021). After reviewing the evidence, they elaborated a questionnaire with various statements and questions about the management of patients with COVID-19 and diabetes/hyperglycemia, grouped into 5 topics: (1) Risk factors for and screening of malnutrition; (2) Assessment and diagnosis of malnutrition; (3) Energy and nutrient requirements; (4) Enteral nutritional care plan (oral and feeding tube); (5) Malnutrition reevaluation and monitoring.

The scientific committee answered remotely the questionnaire and rated their agreement with each statement on a 5-point Likert scale (1—strongly disagree, 5—strongly agree) [22]. A cut-off of 80% or more agreement among them determined consensus. Those questions that did not obtain the stablished degree of agreement were discussed in a virtual meeting to try to understand the reasons of no consensus and/or try to reach consensus. The experts then generated the recommendations for nutritional management of hospitalized COVID-19 patients with diabetes/hyperglycemia, which were considered the optimal care.

### Daily practice reported by the panel of physicians

A selected sample of 90 physicians with more than 5 years of experience and treating COVID-19 patients were sent the questionnaire elaborated by the coordinators to gather their opinions and usual practice regarding each of the topics addressed in the consensus recommendations. The agreement with each statement was again rated by the Likert scale [22], and they were allowed to explain the reasons of agreement or disagreement as free-text comments. The responses of the panel of physicians (“panelists”) were compared with the clinical management recommended by the experts. To facilitate the comparison, each recommendation is described together with the corresponding clinical practice reported by the panelists in tabular format.

## Results

The most frequent medical specialty among the 90 panelists was Endocrinology (72.2%), followed by Geriatrics (12.2%) and Internal Medicine (10.0%).

### Risk factors for and screening of malnutrition

The questionnaire began with a series of questions about the characteristics of patients with diabetes/hyperglycemia and COVID-19 and the associated risk factors for adverse outcomes. As reflected by the answers of the panelists, most of them (87.8%) agreed that diabetes mellitus is one of the most common comorbidities in COVID-19 patients; according to their experience, the presence of hyperglycemia is a poor prognosis factor for these patients. Hospitalized diabetic patients with COVID-19 are often at risk of malnutrition, and they estimated that nearly 50% of the patients cared by them had malnutrition. All were aware that obesity in patients with diabetes/hyperglycemia could mask the risk of malnutrition, and they agreed that patients admitted for SARS-CoV-2 infection are at high risk of malnutrition; 95% of respondents believed that dyspnea and anorexia are symptoms that can increase the risk of malnutrition by compromising a sufficient intake, whereas 87% indicated diarrhea as a symptom that could increase the risk of malnutrition, due to poor nutrient absorption.

Table 1 shows the consensus recommendations reached by the scientific committee related to the risk factors for and screening of malnutrition, and the responses of the panelists for each statement. Panelists’ answers indicated that only 1 out 3 used a severity scale in patients with COVID-19, being the CURB-65 severity scale [23] the most common. For nutritional screening, the Malnutrition Universal Screening Tool (MUST) [24] was the preferred both by the experts and the panelists. The nutritional assessment should be based on diet survey, analytical parameters, and anthropometrics, although other measures are also useful. GLIM 2019 criteria [27] for diagnosis of malnutrition were recommended and the predominantly used.

**Table 1:** Comparative of the optimal care according to the experts’ recommendations and the usual clinical practice of the panelists regarding risk factors for and screening of malnutrition

Risk factors for and screening of malnutrition in patients with diabetes/hyperglucemia and covid-19 infection	
Expert Statement/Recommendation	Panelists’ responses
1. It is essential to identify those patients with COVID-19 who are at high risk of adverse clinical outcomes, so that they can be closely monitored and early intervened upon health deterioration. For this purpose, they recommended using the CURB-65 severity scale [23], useful both in the initial decision to hospitalize and in support of clinical judgment	32% of panelists informed using a severity scale; of them, 69% used the CURB-65 severity score
2. Nutritional screening is recommended for all patients with diabetes/hyperglycemia and COVID-19 at hospital admission. The most suitable tools are the Malnutrition Universal Screening Tool (MUST) [24] and the Remote–Malnutrition APP (R-MAPP) [25], which combines nutritional screening with MUST and sarcopenia screening with the SARC-F [26]	The most common tools for the screening were MUST (70.8%) and MNA-short form (50.6%). 96.6% of panelists declared to perform the nutritional screening just after hospital admission
3. At the same time of screening, a nutritional assessment should be performed for those patients detected at risk or with malnutrition. The recommended tools are (ordered by preference): - Diet survey - Analytical parameters: albumin, CRP and HbA1C - Anthropometric parameters: BMI, weight, height, and percentage of usual weight - Hand-grip strength by dynamometry - Body composition (bioimpedance)	92% carry out the nutritional assessment at hospital admission, mainly together with the nutritional screening. The most useful measures considered were analytical parameters, anthropometrics, and diet survey
4. The diagnosis of malnutrition should be based on the GLIM 2019 criteria [27]. At least one phenotypic and one etiological criteria should be met to confirm the diagnosis of DRM	The predominant criteria for setting the diagnosis of malnutrition were those of GLIM 2019 (90.9%)

*CRP* C-reactive protein, *GLIM* Global Leadership Initiative on Malnutrition, *MNA* Mini Nutritional Assessment, *SARC-F* Strength, assistance with walking, rising from a chair, climbing stairs, and falls questionnaire

### Energy and nutrient requirements

As showed in Table 2, the calculation of energy expenditure and establishing energy and protein requirements in patients without renal failure had a high degree of agreement. For patients with renal failure, protein needs were more controverted. The panelists reported that the most important diet components among the recommended by the experts were proteins of high biological value, low glycemic index and slow absorption carbohydrates; polyunsaturated fatty acids with anti-inflammatory properties, and specific amino acids (leu) or their metabolites (β-hydroxy-β-methylbutyrate).

**Table 2:** Comparative of the optimal care according to the experts’ recommendations and the usual clinical practice of the panelists regarding energy and nutrient requirements

Energy and nutrient requirements in patients with diabetes/hyperglucemia and covid-19 infection	
Expert Statement/Recommendation	Panelists’ responses
5. To calculate the specific energy expenditure of patients, it is recommended to use estimation formulas according to body weight. When BMI > 30 kg/m^2^, the adjusted body weight (BW) should be used (Adjusted BW = Ideal BW + 0.25 x [actual BW – ideal BW]) [27]	86.4% agreed with the recommendation
6. The energy requirements of hospitalized COVID-19 patients with diabetes/hyperglycemia are 25–30 kcal/kg body weight/day and protein requirements are 1.2 g/kg body weight/day. For those patients with renal failure, protein needs range from 0.8 g/kg body weight/day in pre-dialysis to 1.2–1.5 g/kg body weight/day in renal replacement therapy	73% of panelists stated their agreement with the caloric provision of 25–30 kcal/kg body weight/day, whereas 90% indicated that the protein requirements that they use are 1.2–2.4 g/kg body weight/day. For pre-dialysis and renal replacement therapy, the protein requirements were set in 0.8 g/kg body weight/day (54.4%) and 1.2–1.5 g/kg body weight/day (~ 55%) respectively
7: The following components should be included for intake: - Proteins of high biological value (casein, whey, soy) - Specific amino acids (leu) or their metabolites (β-hydroxy-β-methylbutyrate) - Fats: monounsaturated fatty acids, polyunsaturated fatty acids with anti-inflammatory properties (EPA, DHA) - Fiber (fructooligosaccharides) - Low glycemic index and slow absorption carbohydrates (maltodextrins) - Liquids - Vitamins and trace elements (calcium, vitamin D)	Percentages of agreement: 94.4% for proteins of high biological value; 83.3% for specific amino acids 87.8% for polyunsaturated fatty acids with anti-inflammatory properties; 78.9% for fiber 90.0% for low glycemic index and slow absorption carbohydrates; 68.9% for liquids; 78.9% for vitamins and trace elements

*DHA* Docosahexaenoic acid, *EPA* Eicosapentaenoic acid

### Enteral nutritional care plan

A specific diabetes diet for patients with diabetes/hyperglycemia and COVID-19 seems mandatory. The experts recommended the glycemic goals established by the American Diabetes Association; however, the panelists mostly preferred a fasting glycemia below 140 mg/dL The postprandial glucose below 180 mg/dL had a higher level of agreement (Table 3). There was a general agreement on the adequacy of supplementing with a diabetes-specific polymeric, hypercaloric and high-protein formula for patients with diabetes/hyperglycemia and COVID-19, as this formula can achieve an adequate glycemic control. When oral intake is impaired, the best option is a nasogastric tube (NGT) for enteral feeding. Agreeing with the experts’ recommendation, most of the panelists opined that in case of risk of aspiration or limited gastric tolerance despite treatment with prokinetic drugs, a nasojejunal tube (NJT) may be placed for enteral nutrition. For prolonged tube enteral nutrition (> 4 weeks), gastrostomy was considered as an adequate route of administration (Table 3). Half of the panelists used formulas for tube feeding with the same characteristics as the oral nutrition supplements, and the other half of them used specific formulas for enteral tube feeding. The speed of feeding should be progressive to facilitate tolerance.

**Table 3:** Comparative of the optimal care according to the experts’ recommendations and the usual clinical practice of the panelists regarding the enteral nutritional care plan

Enteral nutritional care plan in patients with diabetes/hyperglucemia and covid-19 infection	
Expert Statement/Recommendation	Panelists’ responses
8. Oral feeding is the main choice for all patients, as long as the nutritional requirements are met. A specific diabetes diet is recommended from admission, with aliments easy to ingest and good flavored, adapted in texture for those patients who need it, and adapted in fiber if there are gastrointestinal symptoms. Glycemic goals and monitoring in these patients will be based on those recommended by the American Diabetes Association [28]: - Glycemic goal: 140–180 mg/dL (may be 110–140 mg/dL in selected patients) - Postprandial glucose: < 180 mg/dL Continuous glucose monitoring (CGM) is advisable to minimize contact between health care providers and patients, especially those in the intensive care unit	71% of panelists always establish a specific diet for patients with diabetes/hyperglycemia and COVID-19 just after admission. Among the participants who established a specific diet, 71% considered that the diet adaptation should be based on enriched aliments, and 69% that it should be texture-modified The most fasting glycemic goal selected by the panelists was < 140 mg/dL (41.1%), whereas for postprandial glucose was < 180 mg/dL (64.5%)
9. Depending on the intake and the nutritional assessment during hospitalization, patients’ diets should be supplemented with a diabetes-specific polymeric, hypercaloric and high-protein formula, since these products achieve adequate glycemic control. In the event that the patient cannot ingest 50% of the hospital diet or nutritional supplementation for more than 24 h, it is recommended to start enteral nutritional support	About 84% of panelists chose a diabetes-specific polymeric, hypercaloric and high-protein composition for patients with diabetes/hyperglycemia and COVID-19, and they opined that this formula can achieve an adequate glycemic control Most of the panelists (70.4%) stated that they initiate the enteral nutritional support when the patient cannot ingest 50% of the hospital diet or nutritional supplementation for more than 48 h
10. In case of impeded oral intake, the nasogastric tube (NGT) is the enteral route of choice, provided that there is no added risk of aspiration (i.e., diabetic gastroparesis) or limited gastric tolerance despite treatment with prokinetic drugs. In these cases, a nasojejunal tube (NJT) will be placed for enteral nutrition. If prolonged tube enteral nutrition (> 4 weeks) is expected and the patient no longer has active SARS-CoV-2 infection (negative PCR), gastrostomy should be considered as an indicated route of administration	There was a generalized agreement with these statements: - NJT in case of high risk of aspiration, 79.5%; - NJT in case of limited gastric tolerance despite using prokinetic drugs, 81.8%; - Use of gastrostomy when indicated, 92.0%
11. The formulas for tube enteral nutrition should have the same characteristics as oral nutrition supplements. It is advisable to facilitate tolerance to tube-based feeding by starting with low-dose feeds; volumes of the feeds should be increased in case of good tolerance until reaching the total requirements. Peristaltic pump infusion can improve tolerance versus gravity-fed infusion	51% of the panelists indicated that they used formulas for tube feeding with the same characteristics as the oral nutrition supplements; 49% of them used specific formulas for enteral tube feeding; 93% initiates tube feeding with low-dose feeds; 67.0% used peristaltic pumps
12. For 1st-2^nd^ day of enteral feeding, start feed at 20 mL/h to test tolerance, and increase the speed every 6 h to try to achieve 50% of patients' nutritional requirements in the first 24–48 h. At days 3 to 7, increase progressively, assessing digestive tolerance, to 20–25 kcal/kg/day. From day 7, escalate to 30 kcal/kg/day	Responses of the panelists were: Days 1–2: start feed at 20 mL/h to test tolerance and increase the speed every 6–12 h to try to achieve 50% of patients' nutritional requirements within the first 24–48 h (71.9%); Days 3–7: increase progressively, assessing digestive tolerance, to 20–25 kcal/kg/day (55%); From day 7, escalate to 30 kcal/kg/day (63.3%)

*PCR* polymerase chain reaction

### Malnutrition reevaluation and monitoring

Before hospital discharge, it is recommended to carry out a new nutritional assessment; if the patient has any degree of malnutrition, the nutritional supplementation regimen with hypercaloric and high-protein formulas specific for diabetes/hyperglycemia should be maintained. The panelists opined that the frequency of follow-up visits should depend on the nutritional status of each patient. During the follow up, the most important goal reported by the panelists was to avoid marked fluctuations in blood glucose. Table 4 shows the consensus recommendations reached by the scientific committee related to malnutrition reevaluation and monitoring, and the responses of the panelists for each statement.

**Table 4:** Comparative of the optimal care according to the experts’ recommendations and the usual clinical practice of the panelists regarding malnutrition reevaluation and monitoring

Reevaluation and monitoring of malnutrition in patients with diabetes/hyperglucemia and covid-19 infection	
Expert Statement/Recommendation	Panelists’ responses
13. Before hospital discharge, it is recommended to carry out a new nutritional assessment and establish a monitoring plan according to the status of each patient. If the patient is malnourished, it is necessary to maintain the nutritional supplementation regimen for 3 months, with the hypercaloric and high-protein formulas specific for diabetes/hyperglycemia The goals of the follow up are to maintain or achieve adequate levels of glucose, glycated hemoglobin (within 3 months) and blood lipid profile as soon as possible, and to avoid marked fluctuations in blood glucose	All panelists agreed on scheduling the follow-up visits according to the nutritional status of the patient The most important goals of the follow up were: - Avoid marked fluctuations in blood glucose (91.0%) - Glycemic control (87.6%) - Adequate lipid profile (79.8%) - Achieve adequate levels of glycated hemoglobin (within 3 months) (76.4%)
14.: During patients’ follow up, taking into account that there is no longer risk of contagion, it is recommended to use the following tools for nutritional assessment: - Diet survey - Analytical parameters: albumin and HbA1c - Anthropometric parameters: weight, height, BMI and percentage of usual weight - Dynamometry - Body composition (bioimpedance) In those tests that had altered values, it is important to check if there has been or not an improvement	The percentages of agreement with the recommended tools were: - Analytical parameters: HbA1c (87.6%) and albumin (86.5%) - Anthropometric parameters: BMI (82.0%), weight (80.9%), height (69,7%), and percentage of usual weight (40.4%) - Dynamometry (50.6%) - Body composition (bioimpedance) (42.7%)

## Discussion

The medical community faces an unprecedented pandemic context in which patients’ conditions are diverse and, on many occasions, complicated by comorbidities. Evidence shows that comorbidities, including diabetes, complicate the prognosis of people with COVID-19 [29], and their management poses a relevant clinical challenge. Thus, in this work 18 experts reviewed the evidence, discussed and generated consensus recommendations for the nutritional management of COVID-19 patients with diabetes/hyperglycemia during their hospitalization and after their discharge, and this consensus was contrasted with the usual clinical practice of health professionals (panelists) throughout Spain. A peculiarity of people with diabetes is that they often have a series of risk factors for malnutrition inherent to their disease. In addition to other comorbid conditions and polypharmacy, these patients may suffer from changes in appetite, limited mobility, social isolation, and depression, which contribute to a poor nutritional status and increase the prevalence of malnutrition in this population [30]. In fact, as reported by the panelists, the prevalence of malnutrition in patients with diabetes and COVID-19 was 50%. Importantly, many people with type 2 diabetes are overweight or obese, which can mask the suspicion of malnutrition [31]. However, when asked about this condition, all the panelists were aware that obese patients can actually suffer from malnutrition. Moreover, high BMI has been shown to be significantly associated with critical illness in patients with COVID-19 [32], being the excess adipose tissue the most probable link between obesity and COVID-19 severity [33]. These findings support that those inpatients with abdominal obesity should be monitored even more carefully.

An early determination of the nutritional status is essential to rapidly implement the correct dietetic and supplementary measures, necessary to fight DRM. The experts recommended the MUST [24] and the R-MAPP [25], both tools for malnutrition screening. The R-MAPP serves to remotely screen for malnutrition and sarcopenia, respectively, in a telemedicine setting, but can be useful when no direct contact with the patient is feasible (patient isolation, containment measures). On their part, the panelists preferred a more classical tool, the Mini Nutritional Assessment (MNA)-short form, that examines possible decreases in food intake, weight loss, psychological stress or acute disease during the past three months, and current mobility, and current BMI [34]. The nutritional assessment should be done, and this was the common practice according to the panelists, at the same time of screening. The assessment mainly relies on the analytical parameters, anthropometrics, and diet survey, although dynamometry and bioimpedance can also be useful [35, 36]. The practical guidance released by the European Society of Parenteral and Enteral Nutrition (ESPEN) recommends the use of GLIM criteria for diagnosing malnutrition in COVID-19 patients [37]. All these nutritional evaluations are time-consuming and required trained personnel to be available, but in the event of hospital collapse/high demand for hospitalization, a number of patients may not be evaluated. If the screening and/or nutritional assessment cannot be carried out, and given that patients with diabetes/hyperglycemia and COVID-19 have high probabilities of presenting or developing malnutrition during hospitalization, assuming malnutrition may be an appropriate approach [38]. Even more, it may be advisable to screen and monitor glycemic and nutritional status during the routine evaluation of all COVID-19 patients, as elevated blood glucose levels predicted worse outcomes in hospitalized patients [39].

Sufficient provision of energy and protein is essential for improving clinical outcomes [40]. The energy and protein requirements for hospitalized COVID-19 patients with diabetes/hyperglycemia are based on the ESPEN recommendations [37], and most the panelists agreed on these estimations. However, protein requirements for patients with renal failure were deemed insufficient by the panelists, possibly because an update of the ESPEN recommendations [41] was published on the time interval between the experts' consensus and the panelist survey. These guidelines stated that, in hospitalized patient with acute kidney insufficiency and/or chronic kidney disease with acute/critical illness, not on renal replacement therapy, we should start with 1 g/kg body weight/day, and gradually increase up to 1.3 g/kg body weight/day if tolerated. Patients on renal replacement therapy require higher protein intakes (1.3–1.5 g/kg/d) [41].

Inpatients with SARS-CoV-2 infection deal with particular barriers for a correct intake, due to the need for isolation. This means that they cannot count on external help (for example, a family member) during hospitalization, which can further increase the risk of malnutrition. Therefore, it is essential that the patients are aware of the relevance of a good diet/nutrition to accelerate their recovery and to provide them an adapted and enriched diet to favor intake [42]. An important goal is to achieve or maintain adequate glycemic levels, as recommends the American Diabetes Association [28]. Oral nutritional supplements could be prescribed to meet patient's needs, being the best option a diabetes-specific polymeric, hypercaloric and high-protein formula according to the experts’ and panelists’ opinions. These supplements were deemed appropriate to maintain an adequate glycemic control. Apart from covering energy and protein requirements, there are other components of the diet that may play a relevant role in COVID-19 severity. This is the case of vitamin D, which has shown an association between its deficiency and severity/mortality of COVID-19 [43, 44], highlighting the need for avoiding hypovitaminosis D. When oral intake is not possible, NGT or NJT tubes can be inserted for enteral nutrition, depending on the presence of diabetic gastroparesis or limited gastric tolerance; gastrostomy is indicated when prolonged enteral nutrition is expected [45].

It has been shown that COVID-19 patients who lose more than 10% of their habitual weight during their hospital stay or are admitted to an ICU present a high nutritional risk during follow-up [46]. The group of experts and the panelists agreed on the necessity of a new nutritional assessment before hospital discharge and to maintain the diabetes-specific oral supplements if patients are still malnourished. An important goal during nutritional monitoring is to avoid marked fluctuations in blood glucose, which can be exacerbated by COVID-19 [47], although the glycemic control and achieving a good lipid profile are also important.

In conclusion, the recommendations proposed by this expert group are intended to provide a useful guide on the clinical management of malnutrition in patients with COVID-19 and diabetes/hyperglycemia, in order to improve their outcomes and accelerate as much as possible their recovery. The comparison of the recommended optimal care with routine clinical practice makes it possible to identify gaps in knowledge, implementation difficulties, and areas for improvement in the management of malnutrition in this population.

## Abbreviations

COVID-19: Coronavirus disease 2019
SARS-CoV-2: Severe acute respiratory syndrome coronavirus 2
DRM: Disease-related malnutrition
MUST: Malnutrition Universal Screening Tool
GLIM: Global Leadership Initiative on Malnutrition
CRP: C-reactive protein
MNA: Mini Nutritional Assessment
SARF-C: Strength, assistance with walking, rising from a chair, climbing stairs, and falls
BMI: Body mass index
BW: Body weight
EPA: Eicosapentaenoic acid
DHA: Docosahexaenoic acid
NGT: Nasogastric tube
NJT: Nasojejunal tube
PCR: Polymerase chain reaction
HbA1c: Hemoglobin A1c
R-MAPP: Remote Malnutrition APP
ESPEN: European Society of Parenteral and Enteral Nutrition
CGM: Continuous glucose monitoring
